# The Incretin Effect of Yerba Maté (*Ilex paraguariensis*) Is Partially Dependent on Gut-Mediated Metabolism of Ferulic Acid

**DOI:** 10.3390/nu17040625

**Published:** 2025-02-09

**Authors:** Elijah T. Cooper-Leavitt, Marley J. Shin, Colson G. Beus, Alden T. Chiu, Genevieve Parker, Jack H. Radford, Ethan P. Evans, Isaac T. Edwards, Juan A. Arroyo, Paul R. Reynolds, Benjamin T. Bikman

**Affiliations:** Department of Cell Biology and Physiology, Brigham Young University, Provo, UT 84602, USA

**Keywords:** yerba maté, glucagon-like peptide-1, gastric inhibitor peptide, incretin, bioactive molecules, ferulic acid, dihydroferulic acid, incretins, gut hormones

## Abstract

Background/Objectives: Yerba maté (YM), a traditional herbal beverage made from *Ilex paraguariensis*, contains bioactive compounds like polyphenols and alkaloids known for their metabolic benefits. This study investigates YM’s incretin effects, focusing on glucagon-like peptide-1 (GLP-1) and gastric inhibitory polypeptide (GIP). **Methods**: Male and female C57BL/6 mice were supplemented with YM for four weeks. Post-supplementation, GLP-1 and GIP gene expression levels were analyzed in jejunal mucosa, and plasma hormone concentrations were measured. Additionally, in vitro experiments were conducted using GLUTag L-cells to evaluate the direct effects of YM and its metabolites, including ferulic acid and dihydroferulic acid, on GLP-1 secretion. Gene expression analysis involved quantitative real-time PCR, while hormone levels were assessed via ELISA. Results: YM supplementation significantly increased GLP-1 gene expression and plasma GLP-1 levels compared to controls, with no changes observed in GIP expression or plasma levels. Direct treatment of GLUTag L-cells with YM did not enhance GLP-1 secretion. However, dihydroferulic acid, a microbial metabolite of ferulic acid, markedly stimulated GLP-1 production in L-cells, highlighting a role of gut-mediated metabolism in YM’s incretin effects. Conclusions: YM selectively upregulates GLP-1 pathways without affecting GIP, likely through gut-mediated mechanisms. These findings suggest YM as a promising nutraceutical for incretin modulation and metabolic disorder management. Further studies should explore the interplay between YM, the gut microbiota, and incretin pathways to fully realize its therapeutic potential.

## 1. Introduction

Incretins are a class of metabolic hormones that play a pivotal role in glucose homeostasis, appetite regulation, and overall energy balance. The two major incretins, glucagon-like peptide-1 (GLP-1) and gastric inhibitory polypeptide (GIP), are secreted from the intestinal enteroendocrine cells in response to nutrient intake. GLP-1, secreted predominantly by L-cells in the distal intestine, enhances glucose-dependent insulin secretion, delays gastric emptying, and promotes satiety. Conversely, GIP, produced by K-cells in the proximal intestine, also stimulates insulin secretion but lacks the satiety effects observed with GLP-1. Dysregulation of incretin function has been implicated in the pathogenesis of metabolic disorders such as obesity and type 2 diabetes mellitus (T2DM) [1]. The identification of compounds that modulate incretin levels has, therefore, become a key area of metabolic research.

Dietary bioactives derived from natural products have demonstrated significant promise in regulating metabolic pathways, including incretin secretion. Yerba maté (*Ilex paraguariensis*), a traditional herbal infusion widely consumed in South America, is recognized for its rich composition of secondary metabolites such as polyphenols, flavonoids, alkaloids, and saponins [2]. Several studies have reported its antioxidant, anti-inflammatory, and lipid-lowering properties, suggesting potential applications in managing metabolic disorders [3,4,5]. For instance, yerba maté consumption has been associated with improved lipid profiles, reduced oxidative stress, and weight loss in both preclinical and clinical studies [6,7]. However, the specific effects of yerba maté on incretin modulation, particularly GLP-1 and GIP, remain underexplored.

Incretin-targeted therapies have become a cornerstone of T2DM management, with GLP-1 receptor agonists demonstrating robust efficacy in improving glycemic control and promoting weight loss [1,8,9]. However, these pharmacological interventions often involve high costs and may induce adverse effects, such as gastrointestinal disturbances. As such, alternative approaches, including nutraceutical interventions such as the use of yerba maté, offer a potentially safer and more accessible alternative for modulating incretin pathways. Recent studies indicate that yerba maté consumption can prevent weight gain, enhance mitochondrial efficiency, and improve redox balance, suggesting systemic metabolic benefits [10]. Despite these promising findings, the underlying mechanisms, particularly those involving incretin hormones, require further elucidation.

Among myriad potential benefits to yerba maté, several polyphenols have received attention [2]. Emerging evidence suggests that the gut microbiota plays a central role in mediating the metabolic effects of dietary polyphenols [11,12]. Many plant-derived bioactives, including phenolic acids and flavonoids, are poorly absorbed in the upper gastrointestinal tract and are metabolized by gut microbiota into bioactive derivatives. These metabolites often exhibit enhanced biological activity compared to their parent compounds [13]. This suggests the possibility that some native polyphenols, while being active, may be of greater benefit to the body after undergoing some degree of microbiome-mediated metabolism.

In this study, we hypothesize that the incretin-modulating effects of yerba maté are mediated, at least in part, by the gut microbiome. Specifically, we aim to determine whether yerba maté supplementation enhances GLP-1 and GIP secretion in vivo and to identify key bioactive metabolites responsible for these effects. By combining in vivo and in vitro approaches, this work seeks to advance our understanding of the metabolic benefits of yerba maté and its potential as a nutraceutical intervention for obesity and T2DM.

## 2. Methods

### 2.1. Animals and Diets

Male and female C57BL/6 mice (six months old) were housed at standard conditions (i.e., 22 ± 1 °C, 60–70% humidity, 12 h light–dark cycle) with ad libitum access to food (LabDiet 5001, LabDiet, St. Louis, MO, USA). Mice were randomly divided into two groups (n = 6, three of each sex) and given free access to either water (control, CON) or yerba maté (MATÉ; provided by Unicity International, Provo, UT, USA) for four weeks. The yerba maté extract was prepared by spray drying an aqueous extract of *Ilex paraguariensis* leaves and was provided to the animals per the manufacturer’s instructions (7.49 g yerba maté powder per 300 mL water). Daily drink consumption was monitored and mice were weighed weekly. 

Studies were conducted in accordance with the principles and procedures outlined in the National Institutes of Health Guide for the Care and Use of Laboratory Animals and were approved by the Institutional Animal Care and Use Committee at Brigham Young University (protocol #20-0203).

### 2.2. Sample Collection

At the end of the treatment period, mice were fasted for 6 h and then euthanized by CO_2_ inhalation. Blood samples were collected via cardiac puncture into EDTA-coated tubes containing dipeptidyl peptidase IV inhibitor (MilliporeSigma, Burlington, MA, USA) to prevent GLP-1 degradation. Plasma was separated by centrifugation at 4 °C and stored at −80 °C until analysis. The jejunum was excised and rinsed with ice-cold saline, and the mucosal layer was scraped using a glass slide. Scraped tissues were snap-frozen in liquid nitrogen and stored at −80 °C for subsequent RNA extraction.

### 2.3. Gene Expression Analysis

Total RNA was extracted from jejunal mucosa using the RNeasy Mini Kit (Qiagen, Hilden, Germany) following the manufacturer’s instructions. cDNA synthesis was performed using the High-Capacity cDNA Reverse Transcription Kit (Applied Biosystems, Foster City, CA, USA). Quantitative real-time PCR was conducted using TaqMan Gene Expression Assays (Applied Biosystems) specific for GLP-1 (Gcg) and GIP (Gip) genes, with β-actin (Actb) serving as the endogenous control. Relative gene expression levels were calculated using the 2^−ΔΔCt method.

### 2.4. Plasma Incretin Measurements

Plasma concentrations of active GLP-1 and total GIP were measured using commercially available ELISA kits (MilliporeSigma; Burlington, MA, USA) according to the manufacturer’s protocols. All samples and standards were assayed in duplicate.

### 2.5. Cell Culture

GLUTag cells, a murine L-cell line, were kindly provided by Dr. Fiona Gribble (University of Cambridge, UK). Cells were maintained in high-glucose Dulbecco’s Modified Eagle Medium (DMEM; Thermo Fisher Scientific, Waltham, MA, USA) supplemented with 10% fetal bovine serum (FBS; Thermo Fisher Scientific; Pittsburgh, PA, USA), 100 U/mL penicillin, and 100 μg/mL streptomycin at 37 °C in a humidified atmosphere of 5% CO_2_. Cells were passaged at 70–80% confluence using 0.25% trypsin-EDTA.

### 2.6. Treatment Protocols

For treatment experiments, GLUTag cells were seeded in 24-well plates at a density of 1 × 10^5^ cells. Following adherence, cells were incubated with yerba maté extract (0.1–1 mg/mL), ferulic acid (100 μM; MilliporeSigma; Burlington, MA, USA), or dihydroferulic acid (100 μM; Biosynth, Staad, Switzerland) for 24 h. Control cells received vehicle treatment (0.1% DMSO).

### 2.7. GLP-1 Secretion Assay

Following treatment, the culture medium was collected and centrifuged to remove cellular debris. GLP-1 levels in the supernatant were quantified using a GLP-1 (Active) ELISA kit (MilliporeSigma; Burlington, MA, USA) according to the manufacturer’s instructions. Data were normalized to total cellular protein content, determined by bicinchoninic acid (BCA) assay (Thermo Fisher Scientific). All samples were assayed in duplicate.

### 2.8. Statistical Analysis

Data are presented as mean ± standard error of the mean (SEM). GraphPad Prism 8.0 (GraphPad Software, San Diego, CA, USA) was used for statistical analyses. Comparisons between groups were made using unpaired Student’s *t*-tests or one-way ANOVA followed by Tukey’s post hoc test, as appropriate. A *p*-value < 0.05 was considered statistically significant.

## 3. Results

### 3.1. Yerba Maté Enhances GLP-1 Gene Expression in the Jejunum and Plasma Protein Levels

**Figure 1:**
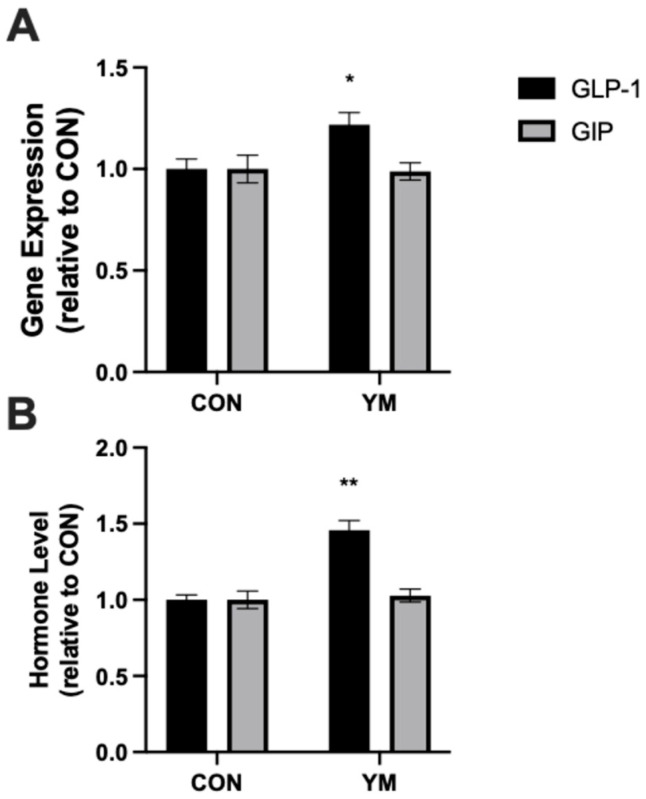
Effects of yerba maté (YM) supplementation on jejunal gene expression and plasma hormone levels. (**A**) GLP-1 and GIP gene expression in the scraped jejunum of control (CON) and YM-treated mice, expressed relative to CON as fold change. (**B**) Plasma levels of active GLP-1 and total GIP in CON and YM-treated mice, expressed relative to CON. Data are presented as mean ± SEM. * *p* < 0.05 vs. CON, ** *p* < 0.01 vs. CON.

To evaluate the effect of yerba maté (YM) supplementation on incretin gene expression, GLP-1 and GIP mRNA levels were quantified in jejunal mucosa from control (CON) and YM-treated mice. GLP-1 mRNA expression was significantly upregulated in YM-treated mice compared to controls (Figure 1A, *p* < 0.05). In contrast, no significant difference in GIP gene expression was observed between the groups.

Consistent with the upregulation of GLP-1 gene expression, plasma levels of active GLP-1 were significantly higher in YM-treated mice compared to controls (Figure 1B, *p* < 0.01). However, plasma levels of GIP remained unchanged between the two groups.

### 3.2. Yerba Maté Does Not Directly Stimulate GLP-1 Production in L-Cells

**Figure 2:**
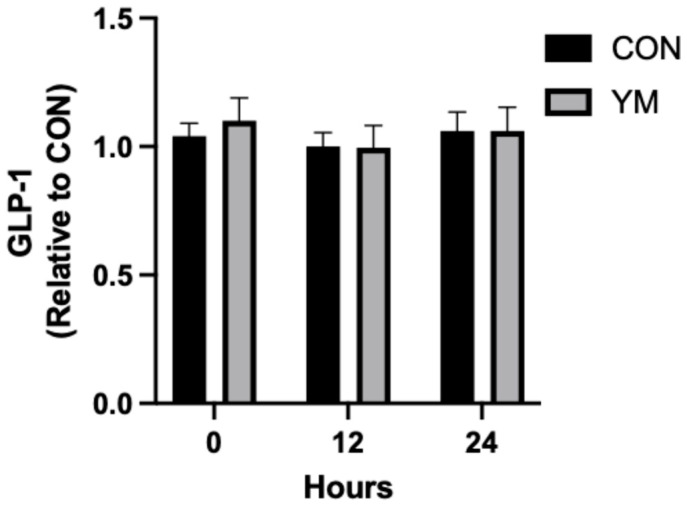
Time course of GLP-1 secretion in L-cells treated with yerba maté (YM). GLUTag cells were incubated with YM or vehicle (CON) for 0, 12, and 24 h, and GLP-1 levels in the supernatant were quantified. Data are presented as mean ± SEM and expressed relative to CON as fold change. No significant differences were observed at any time point.

To assess the direct effects of YM on GLP-1 secretion, GLUTag L-cells were incubated with YM for 0, 12, and 24 h, and GLP-1 levels in the supernatant were quantified. No significant changes in GLP-1 secretion were observed at any time point compared to the untreated controls (Figure 2). These data suggest that YM does not directly stimulate GLP-1 production in L-cells and may require alternative pathways or intermediates to exert its effects.

### 3.3. Gut-Mediated Changes Enhance GLP-1 Secretion

**Figure 3:**
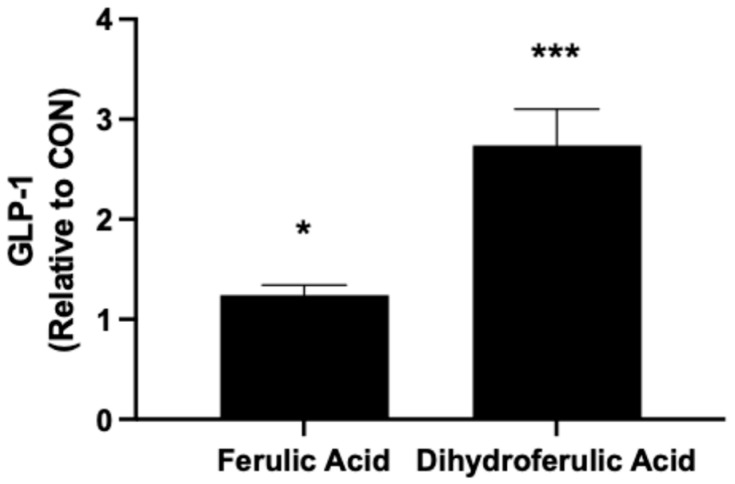
Effects of ferulic acid and dihydroferulic acid on GLP-1 secretion in L-cells. GLUTag cells were treated with ferulic acid or its microbial metabolite, dihydroferulic acid, for 24 h, and GLP-1 levels in the supernatant were quantified. Data are presented as mean ± SEM and expressed relative to CON as fold change. * *p* < 0.05 vs. CON, *** *p* < 0.001 vs. CON.

Given the potential role of gut microbiota in YM metabolism, ferulic acid and its microbial metabolite, dihydroferulic acid, were tested for their effects on GLP-1 secretion in GLUTag cells. While ferulic acid modestly increased GLP-1 secretion relative to controls (*p* < 0.05), dihydroferulic acid significantly enhanced GLP-1 production by over 2.5-fold (*p* < 0.001; Figure 3). These results support the hypothesis that YM’s effects on GLP-1 are mediated by microbial metabolism, with dihydroferulic acid playing a critical role in incretin modulation.

## 4. Discussion

This study builds on our previous work demonstrating the metabolic benefits of yerba maté (YM), providing novel insights into its mechanisms of action, particularly its ability to modulate incretin levels. Here, we show that YM supplementation selectively enhances GLP-1 gene expression and plasma levels without affecting GIP, and propose a gut microbiota-mediated mechanism as a key driver of these effects. These findings complement earlier observations from our laboratory, which highlighted the tissue-specific effects of YM on mitochondrial efficiency and redox balance [10]. Importantly, throughout all outcomes, no differences were observed between the sexes.

### 4.1. Yerba Maté Enhances GLP-1 Without Affecting GIP

The selective upregulation of GLP-1 gene expression and plasma levels observed in YM-treated mice is consistent with the broader metabolic benefits of GLP-1, including improved glycemic control, enhanced insulin secretion, and appetite regulation [14,15]. Unlike GIP, which primarily promotes insulin secretion, GLP-1 offers additional therapeutic advantages, such as delayed gastric emptying and increased satiety [8]. By selectively targeting GLP-1 without increasing GIP, YM may avoid the lipogenic effects associated with elevated GIP levels [16].

Our findings align with previous studies from our laboratory, which showed that YM supplementation prevents weight gain and improves mitochondrial efficiency in skeletal muscle while reducing mitochondrial efficiency in adipose tissue—a pattern indicative of metabolically beneficial tissue-specific effects [10]. These effects may contribute to the observed GLP-1 upregulation, as mitochondrial health has been linked to the functionality of enteroendocrine cells and incretin secretion.

### 4.2. Role of Microbial Metabolism

While YM increased GLP-1 levels in vivo, it failed to directly stimulate GLP-1 secretion in L-cells. This discrepancy underscores the role of gut microbiota in metabolizing YM compounds into bioactive derivatives. Ferulic acid, a phenolic compound present in YM, modestly enhanced GLP-1 secretion in L-cells, whereas its microbial metabolite, dihydroferulic acid, robustly increased GLP-1 secretion. This finding is consistent with the growing body of evidence linking gut microbiota transformations of dietary polyphenols to systemic metabolic effects [17].

Previous studies from our lab have demonstrated the critical role of redox balance in mitochondrial and metabolic health, highlighting YM’s ability to modulate hepatic redox potential toward a more reduced state [10]. This effect may enhance intestinal epithelial health, facilitating the gut microbiota’s capacity to metabolize polyphenols into bioactive metabolites. The ability of dihydroferulic acid to significantly stimulate GLP-1 secretion suggests that YM’s metabolic benefits are mediated by microbiome–host interactions, adding another layer to its mechanism of action.

### 4.3. Molecular Mediators of GLP-1 Secretion in YM

Multiple molecules in YM are known to mediate an increase in GLP-1 levels, most especially chlorogenic acid and ferulic acid. Chlorogenic acid is well known for its role in directly stimulating GLP-1 secretion from enteroendocrine L-cells, contributing to enhanced insulin release and improved glycemic control. Studies have demonstrated that chlorogenic acid increases GLP-1 levels in vitro and in vivo, supporting its potential as a natural incretin enhancer [18]. Additionally, it has been shown to modulate gut microbiota and reduce metabolic inflammation, further enhancing GLP-1 signaling [19]. These findings suggest that chlorogenic acid plays a significant role in yerba mate’s metabolic effects through multiple pathways.

In contrast, ferulic acid’s role in GLP-1 regulation remains less well understood, despite its presence in yerba mate and other plant-derived foods. Emerging evidence suggests that ferulic acid primarily exerts its effects by protecting GLP-1 from enzymatic degradation rather than directly stimulating its secretion. Specifically, ferulic acid has been shown to bind GLP-1 and inhibit dipeptidyl peptidase-4 (DPP-4), the enzyme responsible for its rapid inactivation, thereby extending the hormone’s biological activity [20]. By increasing the stability of GLP-1, ferulic acid may enhance its insulinotropic effects and contribute to improved metabolic outcomes. This mechanism distinguishes it from chlorogenic acid, which acts more directly on GLP-1 secretion.

Given the extensive research on chlorogenic acid, our brief study focused on ferulic acid to further explore its contribution to the metabolic effects of yerba mate. The limited understanding of its role in GLP-1 regulation presents an opportunity to expand knowledge on non-nutrient-based modulators of incretin activity. Future studies should investigate whether ferulic acid’s DPP-4 inhibitory effects translate into meaningful metabolic improvements in human populations and whether its actions are synergistic with other bioactive compounds in yerba mate. Understanding the interplay between these molecules may help clarify the potential of yerba mate as a functional food for metabolic health.

### 4.4. Limitations and Future Directions

This study has notable limitations. While we demonstrated the importance of the ferulic acid metabolite dihydroferulic acid in YM’s effects, we did not directly assess microbiota composition or activity. Incorporating microbiome sequencing and metabolomics in future research would provide deeper mechanistic insights. Moreover, the use of a murine model and in vitro L-cell assays limits the generalizability of our findings to humans.

To further elucidate the role of gut microbiota in YM’s effects on GLP-1 secretion, future studies should identify and test specific bacterial strains capable of metabolizing ferulic acid into dihydroferulic acid. Candidate strains, such as those within the genera *Lactiplantibacillus*, *Fructilactobacillus*, and *Enterococcus*, are promising based on their known abilities to reduce ferulic acid to dihydroferulic acid. Experiments using gnotobiotic mice colonized with defined microbial communities or mono-associated with individual strains will allow precise evaluation of their contributions to GLP-1 modulation. Additionally, in vitro co-culture systems combining these bacteria with L-cells could clarify the mechanistic pathways linking microbial metabolism to enteroendocrine function. Advanced approaches, such as CRISPR-based gene editing of bacterial metabolic pathways, could also be employed to optimize dihydroferulic acid production and assess its direct impact on GLP-1 secretion. These investigations will help validate specific microbial strains as potential co-therapeutics for enhancing YM’s metabolic benefits.

## 5. Conclusions

In conclusion, YM selectively enhances GLP-1 gene expression and plasma levels through mechanisms likely mediated by gut microbiota. The robust effects of dihydroferulic acid on GLP-1 secretion highlight the central role of microbial metabolism in YM’s incretin-modulating properties. These findings build on our previous work demonstrating YM’s tissue-specific metabolic effects and underscore its potential as a nutraceutical for managing obesity and T2DM. Future research should explore the interplay between YM, the gut microbiota, and incretin pathways to fully realize its therapeutic potential.

## Data Availability

Data can be available by request.
